# Chromosome-scale genome assembly and developmental transcriptome of *Aedes triseriatus*, vector of La Crosse virus

**DOI:** 10.1186/s12864-026-12779-8

**Published:** 2026-03-25

**Authors:** Erika Nishiduka, Thomas Hill, Brian Bonilla, Lilian Caesar, Paola Valenzuela Leon, Osvaldo Marinotti, Eric Calvo

**Affiliations:** 1 Laboratory of Malaria and Vector Research, National Institute of Allergy and Infectious Diseases, National Institutes of Health, Rockville, MD 20852 USA; 2 Integrated Data Sciences Section, Research Technology Branch, National Institute of Allergy and Infectious Diseases, National Institutes of Health, Bethesda, MD 20892 USA; 3 Department of Biology, Indiana University, Bloomington, IN USA

**Keywords:** Blood-feeding arthropods, Vector-borne diseases, Mosquito, Encephalitis, Functional genomics

## Abstract

**Background:**

*Aedes triseriatus *is an endemic North American mosquito and the primary vector of La Crosse virus, the etiologic agent of La Crosse encephalitis. This neuroinvasive disease is the leading cause of arboviral encephalitis in the United States and disproportionately affects pediatric populations. Despite its medical importance, the genomic architecture and vectorial capacity of *A. triseriatus* remain incompletely understood.

**Results:**

We generated a chromosome-scale genome assembly and developmental transcriptome for *A. triseriatus* using an integrated approach combining PacBio long-read sequencing, Hi-C scaffolding, and Illumina sequencing technologies. The final assembly spans approximately 2.4 Gb, representing the largest mosquito genome reported to date, and consists of chromosome-sized scaffolds supported by Hi-C data and manual curation. Transcriptome profiling across larvae, pupae, and adult males and females revealed dynamic, stage-specific gene expression patterns associated with distinct physiological processes.

**Conclusions:**

These genomic and transcriptomic resources provide a comprehensive foundation for investigating the molecular basis of La Crosse virus transmission, mosquito development, and vector–host interactions in *A. triseriatus*. The chromosome-scale assembly establishes an essential framework for future functional, evolutionary, and vector biology studies in this medically important species.

**Supplementary Information:**

The online version contains supplementary material available at 10.1186/s12864-026-12779-8.

## Introduction

*Aedes triseriatus*, also known by the synonym *Ochlerotatus triseriatus* and commonly known as the eastern tree-hole mosquito, is an endemic species from North America notable for its preference for breeding in small, water-filled natural or artificial containers such as tree holes and discarded tires [1]. Adult females feed mostly on small mammalian hosts, but they are also known to bite humans.

*Aedes triseriatus* is related to the major human disease vector species, *Aedes aegypti*, with an estimated evolutionary separation of 92 million years [2]. Both species are epidemiologically important as vectors of human arboviruses, transmitted when infected female mosquitoes inject pathogens through their saliva during blood feeding [3, 4]. The most significant illness associated with *A. triseriatus* is the pediatric encephalitis caused by the La Crosse virus (LACV), which has been an important cause of arboviral neuroinvasive disease on the continent for over 60 years [5, 6]. Although asymptomatic and underreported in most cases, a small percentage of LACV-infected children develop severe neurological complications, including cognitive impairment and chronic seizures, and infection leads to death in extreme cases [7, 8]. Currently, therapies are only supportive, and vaccines or drugs are not available to prevent this disease [6, 9].

Despite the potential role of *A. triseriatus* in the transmission of both endemic and imported pathogens, the true vectorial capacity of this species, as well as its ability to adapt to specific environmental conditions and eco-ethological contexts, remains poorly understood. A deeper understanding of their basic biology is essential to inform the development of effective mosquito and disease control strategies. However, genomic resources for this species have been limited.

In the literature, the taxonomy of *Aedes triseriatus* has been treated inconsistently, with classifications alternating between *Ochlerotatus* and *Aedes* over the years. This ambiguity stems from the taxonomic revision by Reinert and colleagues, who elevated *Ochlerotatus* and several other taxa from subgenera within *Aedes* to full genera based primarily on morphological characters, substantially expanding the number of recognized aedine genera [10]. Subsequently, Wilkerson et al. [11] reinstated *Aedes* as a single genus comprising multiple subgenera, including *Ochlerotatus*—a framework that has since been widely adopted in medical entomology and vector-control literature. Nonetheless, several online taxonomic inventories and reference sources continue to list *Ochlerotatus* as a valid genus within the tribe Aedini, often noting uncertainty regarding its monophyly and the ongoing debate over whether it should be recognized as a separate genus or maintained as a subgenus of *Aedes*. In accordance with this latter classification, the present study follows the usage of *Aedes* as a single genus and therefore adopting *Aedes triseriatus and Aedes sierrensis* nomenclatures.

Here, we present a chromosome-scale genome assembly supported by Hi-C and a developmental transcriptome data from *A. triseriatus.* These resources presented in this work will inform the subsequent studies aimed at investigating La Crosse virus infection, shedding light on the functional characterization of numerous genes of unknown function and elucidation of their roles in vector-host interactions and pathogen transmission.

## Methods

### Mosquito rearing

The *Aedes triseriatus* mosquitoes were reared under standard insectary conditions (27 °C, 80% humidity, with a 12-h light/dark photo period) at the Laboratory of Malaria and Vector Research, NIAID, NIH. This colony was kindly donated by Dr. Doug E. Brackney (The Connecticut Agricultural Experiment Station, New Haven, CT). Blood feeding was carried out using artificial membrane feeders as described previously by Lu et al. [12].

Eggs from blood-fed females were collected on moist fluted filter paper and maintained under humid conditions for at least two weeks to ensure complete embryonation. Hatching was synchronized by submerging eggs in distilled water followed by vacuum stimulation. Larvae were reared in trays containing 1 L of distilled water and adjusted two days post-hatching to a density of approximately 200 larvae per liter. Larvae were fed TetraMin fish food *ad libitum* until pupation (approximately day 7). Pupae were transferred to distilled water and placed in emergence cages. Adults were maintained on 10% Karo syrup provided on cotton pads. Females aged 4–6 days post-emergence were blood-fed after replacing syrup with water the day prior. Blood meals were delivered using artificial membrane feeders (NDS Technologies, Inc.) covered with Parafilm^®^ M (Amcor) and filled with bovine whole blood in ACD (Lampire Biological Laboratories) supplemented with 100 mM ATP.

### DNA extraction for the PacBio pipeline

Total genomic DNA (gDNA) was extracted from one adult female using the MagAttract HMW DNA Kit (Qiagen, cat. n°. 67563) based on the supplier’s instructions with some modifications (Mullin et al., 2023). Quantification, size determination, and integrity analyses of the extracted gDNA were performed using the Agilent 4200 TapeStation system with Genomic DNA Screentape assays (Agilent Technologies, cat. n°. 5067–5365), following the manufacturer’s instructions.

### PacBio library preparation and sequencing

PacBio library preparation and sequencing was performed at Fredrick National Laboratory for Cancer Research, in the National Institutes of Health (Frederick, MD, USA). The DNA library was sequenced with the PacBio Revio system (Pacific Biosciences, CA). A fraction of 2 µg of the previously eluted gDNA sample was sheared to 14–19 kb in size using the Megaruptor 3 (Diagenode, Inc.) at shearing speed 31. The sheared DNA was purified using 1X SMRTbell cleanup beads and used to prepare whole genome libraries using SMRTbell Prep Kit 3.0 (Pacific Biosciences, CA). The sequencing primers were annealed, and Revio polymerase was bound to the library before loading using the Revio Polymerase kit (Pacific Biosciences, CA). The library was loaded on one Revio SMRTcell at 240 pM loading concentration. Sequencing was performed during a 24-hour movie time on Revio.

### Hi-C library preparation and sequencing

Hi-C sample preparation was conducted by Phase Genomics (Seattle, WA, USA). Library preparation used the Proximo kit version 4.5 (Phase Genomics, Seattle, WA, USA), and sequencing was carried out on an Illumina NovaSeqX Plus 25B flow cell.

Approximately 500 mg of mosquito tissue, derived from 150 adult females, was finely minced, and the DNA was subsequently crosslinked by proximity ligation. The crosslinking reaction was terminated with a quenching solution, and the tissue was rinsed with chromatin rinse buffer. Chromatin was isolated from the sample homogenate using magnetic beads and then fragmented with fragmentation enzyme. The proximity ligation reaction was incubated at room temperature for 4 h, after which free DNA was purified with Recovery Beads, and Hi-C junctions were captured using streptavidin beads. The washed beads were subsequently used to prepare paired-end deep sequencing libraries using the Proximo Library preparation reagents.

### Genome assembly

The generated PacBio long reads were assembled using HifiASM [13], with default parameters including deduplication. Hi-C data were incorporated to improve contiguity and support gap closure and scaffolding. The genome assembly was supplemented with *A. triseriatus* developmental transcriptome data from this study and previously published salivary glands transcriptome and proteome [12]. Following assembly, only haplotype one was retained for downstream analyses. To improve genome contiguity, an intermediate reference-guided scaffolding step was performed in which the *A. triseriatus* assembly was scaffolded against the *Aedes sierrensis* reference genome (GenBank accession ID GCA_044231785.1) using RagTag [14]. *Aedes sierrensis and A. triseriatus* are two distinct but related North American tree-hole mosquito species [15]. To generate a chromosome-scale assembly reflecting *A. triseriatus* chromosomal organization, the resulting scaffolds were manually curated using Juicebox [16]. This curation involved the introduction of 44 breaks across 40 contigs to correct misjoins and refine chromosome structure. Genome assembly and annotation completeness were assessed through BUSCO version. 5.0.0 and Diptera OrthoDB data version 10 (*n* = 3,285 single copy of orthologs).

### RNA isolation

Samples were collected in biological triplicates throughout the *A triseriatus* lifecycle, including larvae, male and female pupae, and male and female adults. Larvae were divided into two groups: Larvae 1, consisting of first and second-instar larvae, and Larvae 2, comprising third and fourth-instar larvae. Pupae and non-blood-fed adults were separated by sex. Blood-fed females were collected at 3, 8, 24, 48, and 72 h post-feeding (Table 1). Samples were manually crushed using sterile pestles in TRIzol (Invitrogen, cat. n° 15596018) and total RNA was extracted following the manufacturer’s instructions.

**Table 1:** Samples used for *A. triseriatus* transcriptome sequencing and gene expression analysis

Sample condition	Group Label	# Specimens per replicate	Collection time point
Larvae, first- and second-instar	L1	15–20	3 days after hatching
Larvae, third- and fourth-instar	L2	5	7 days after hatching
Pupae, male	PM	5	24–48 h after pupation
Pupae, female	PF	5	24–48 h after pupation
Adult male, sugar-fed	M	5	4–6 days after emergence
Adult female, sugar-fed	F0	5	4–6 days after emergence
Adult female, 3 h post blood-feeding	F3	5	4–6 days after emergence
Adult female, 8 h post blood-feeding	F8	5	4–6 days after emergence
Adult female, 24 h post blood-feeding	F24	5	5–7 days after emergence
Adult female, 48 h post blood-feeding	F48	5	6–8 days after emergence
Adult female, 72 h post blood-feeding	F72	5	7–9 days after emergence

### RNA library construction, quality control, sequencing, and analysis

#### NEBNext Ultra II RNA with PolyA Selection

RNA library construction and sequencing was conducted by Novogene (Sacramento, CA, USA). Isolated RNA quality was assessed by RNA Tapestation (Agilent Technologies Inc., California, USA) and quantified by AccuBlue® Broad Range RNA Quantitation assay (Biotium, California, USA). Paramagnetic beads coupled with oligo d(T)25 were combined with total RNA and poly(A)+ transcripts were isolated using the NEBNext® Poly(A) mRNA Magnetic Isolation Module (New England BioLabs Inc., Massachusetts, USA). Before first strand synthesis, samples were randomly primed (5´ d(N6) 3´ [N = A, C,G, T]) and fragmented based on the manufacturer’s recommendations. The first strand was synthesized with the Protoscript II Reverse Transcriptase with a longer extension period, approximately 30 min at 42 °C. All remaining steps for library construction were used according to the NEBNext® Ultra™ II Non-Directional RNA Library Prep Kit for Illumina® (New England BioLabs Inc., Massachusetts, USA). The final library quantity was assessed by Qubit 2.0 (ThermoFisher, Massachusetts, USA), and quality was assessed by TapeStation HSD1000 ScreenTape (Agilent Technologies Inc., California, USA). The final library size was about 480 bp with an insert size of about 330 bp. Illumina® 8-nt dual-indices were used. Equimolar pooling of libraries was performed based on QC values and sequenced on an Illumina NovaSeq X Plus 10B (Illumina, California, USA) with a read length configuration of 150 PE for 40 M PE reads per sample (20 M in each direction).

### Genome annotation

RepeatModeler [17] and RepeatMasker [18] were used to identify repetitive content in the *A. triseriatus* assembly and to softmask the repetitive regions of the genome. We first repeat masked simple repeats, followed by known repeats from RepeatModeler, and subsequently by unknown repeats from RepeatModeler. Maker2 [19] was used to annotate the softmasked assembly, using the reference databases downloaded from the VectorBase server (vectorbase.org, release 67): *Aedes aegypti* LVP_AGWG, *Aedes albopictus* Foshan FPA, and *Culex quinquefasciatus* JHB 2020, in combination with the *de novo* transcriptome database generated from our draft assemblies, which used the Illumina RNA-sequencing for each *A. triseriatus* life stage. Finally, we generated new unique genes and transcript IDs using Maker [20, 21].

### Functional annotation

Blastp (e-value = 0.00001 max, hsps = 1, max, alignments = 1) was applied to identify orthologs among the *Aedes triseriatus* and *Aedes aegypti*,* Aedes albopictus*,* Culex quinquefasciatus* (from vectorbase.org, release 67), *Drosophila melanogaster* (Flybase server, release 6.54, downloaded in December, 2023) and proteins from Uniprot (downloaded in June, 2024). Coding sequences (CDS) were extracted from this custom database search composed of the combination of the three reference databases: *Aedes aegypti* LVP_AGWG, *Aedes albopictus* Foshan FPA, and *Culex quinquefasciatus* JHB 2020, which were downloaded from the VectorBase server (vectorbase.org, release 67).

### Gene and isoform expression

The RNAseek pipeline version 1.9.0 [22] was used to access gene expression. The pipeline assessed the quality of each sample using FastQC v0.11.9 [23], Preseq v2.0.3, Picard tools v2.17.11 [24], FastQ Screen v0.9.3 [25], Kraken2 v2.0.8 [26], QualiMap [27], and RSeQC v2.6.4 [28]. Adapters and low-quality sequences were trimmed using Cutadapt v1.18 [29]. The trimmed reads were merged into a single file and *de novo* assembled with Trinity [30]. Gene and transcript expression levels were estimated against our *A. triseriatus* genome annotation via RSEM v1.3.3 [31]. Results were expressed as transcripts per million (TPM) per gene per sample.

### Mitogenome assembly and annotation, and phylogenetic tree analysis

The *Aedes aegypti* mitochondrial genome and mitochondrial proteins (RefSeq: NC_035159.1) were used as query sequences in BLATSN and BLASTP tools to identify assembled contigs corresponding to the *A. triseriatus* mitochondrial genome. MITOS [32] was applied to annotate the identified mitochondrial genome.

A multispecies alignment of mitochondrial genomes was obtained using MAFFT [33]. Following alignment, we converted the alignment to Phylip format using Biopython [34] and generated a bootstrap whole genome phylogeny with 100 bootstraps using RaxML [35]. We then visualized the final phylogeny in R software using APE [36]. The mitochondrial genomes, from different Diptera species, used in the analysis are listed in the Supplementary Table 1 (Suppl. Table 1).

### Horizontal gene transfer

To identify potential horizontally transferred genes [37–39] in the mosquito genome, we first performed a BLASTP search using DIAMOND v2.1.7.16 against the NCBI non-redundant protein database (nr, version November 11/10/2023), with the following parameters: --max-target-seqs 500, --min-score 50, and --outfmt 6. A custom script (available at https://github.com/liliancaesarbio/alien_hgt_index*)* was then used to assign taxonomic annotations to each BLAST hit using the NCBI Taxonomy database. Based on these annotations, hits were categorized into three taxonomic categories: RECIPIENT (Insecta), GROUP (non-insect Metazoa), and OUTGROUP (non-Metazoan). For each protein, we recorded the e-value and bitscore of the top hit within each lineage: eR and bR (RECIPIENT), eG and bG (GROUP), and eO and bO (OUTGROUP). We then calculated the Alien Index (AI) and HGT Index (HI) for proteins where at least 80% of hits belonged to the OUTGROUP category:

$$\mathrm{Alien}\;\mathrm{Index}\;(\mathrm{AI})\:=\:\log(\mathrm{eG}\:+\:1\mathrm e\:-\:200)\:-\:\log(\mathrm{eO}\:+\:1\mathrm e\:-\:200)$$

$$\mathrm{HGT}\;\mathrm{Index}\;(\mathrm{HI})\:=\:\mathrm{eO}\:-\:\mathrm{eG}$$

Proteins with HI > 45 and either AI > 25 or no hits in the GROUP category were considered candidates for horizontal gene transfer into the mosquito genome. Custom scripts for categorization and calculations are also available at: https://github.com/liliancaesarbio/alien_hgt_index.

## Results

### Genome de novo assembly

**Fig. 1:**
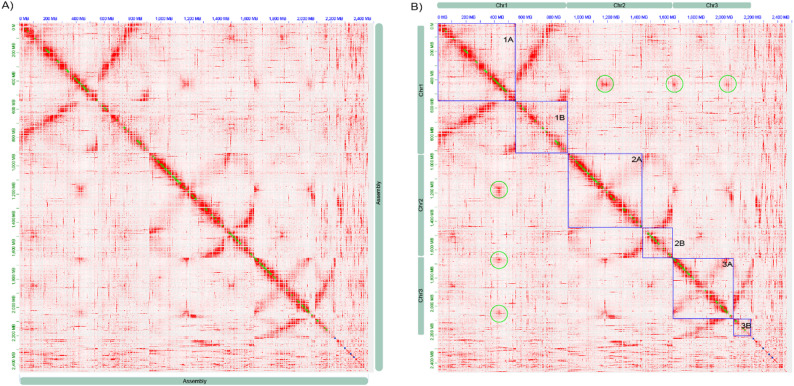
Hi-C contact heatmap of the *Aedes triseriatus* genome. Color intensity represents the frequency of chromatin interactions between genomic loci at a 25-kb resolution. Green dots along the diagonal indicate draft contig boundaries prior to final scaffolding. **a** Genome-wide Hi-C contact map after contig clustering and manual curation. The strong, continuous primary diagonal across each chromosome-sized scaffold supports large-scale scaffold integrity, with distinct arm-level interaction domains visible. **b **Chromosome boundaries (blue boxes) defined by Hi-C interaction patterns. Off-diagonal signals primary reflect interactions between homologous or highly similar repetitive regions rather than true inter-chromosomal interactions. No abrupt interaction discontinuities indicative of large-scale misjoins are observed

Through the integration of PacBio long reads, Hi-C data, and manual curation, we assembled an *A. triseriatus* genome of approximately 2.46 Gb, representing the largest mosquito genome sequenced to date (Table 2; Fig. 1). The read-length distribution histogram (Suppl. Table 2, Suppl. Figure 1), showing a predominance of reads in the 10–20 kb range, indicates that the sequencing data comprised sufficiently long reads to support a contiguous genome assembly.

**Table 2:** Comparative genome statistics of the Aedes genus with reference genomes. Source: NCBI.

	*A. triseriatus*	*A. aegypti*	*A. albopictus*	*A. camptorhynchus*	*A. japonicus*	*A. koreicus*	*A. notoscriptus*	*A. sierrensis*
Database	RefSeq	RefSeq	RefSeq	GenBank/RefSeq	GenBank	GenBank	GenBank	GenBank
Genome size	2.4 Gb	1.3 Gb	1.3 Gb	1.1 Gb	1.4 Gb	1.1 Gb	900.4 Mb	1.2 Gb
Total ungapped length	2.4 Gb	1.3 Gb	1.3 Gb	1.1 Gb	1.4 Gb	1.1 Gb	900.4 Mb	1.2 Gb
Number of chromosomes	3	3	3		25,236	-	3	3
Number of organelles	1	1	1	1	-	1	-	
Number of scaffolds	652	2,309	1,496	1,240	-	6,099	308	366
Scaffold N50	427.4 Mb	409.8 Mb	450.2 Mb	3.3 Mb	118.2 kb	329.6 kb	282 Mb	374.8 Mb
Scaffold L50	2	2	2	97	2,962	896	2	2
Number of contigs	718	2,538	6,081	1,240	25,708	6,126	982	2,771
Contig N50	8.1 Mb	11.8 Mb	1 Mb	3.3 Mb	113 kb	329 kb	1.8 Mb	1.6 Mb
Contig L50	304	30	351	97	3,115	898	150	214
GC percent	38.5	38.5	40.5	40.0	39.5	39.5	40.5	40.0
Genome coverage	11.5x	110.0x	35.0x	44x	20.0x	79.2x	60.0x	10x
Assembly level	Chromosome	Chromosome	Chromosome	Scaffold	Scaffold	Scaffold	Chromosome	Chromosome
Sequencing technology	Hi-C PacBio Revio	PacBio	PacBio Hifi	PacBio Revio	Oxford Nanopore PromethION	Illumina; Oxford Nanopore MinION	PacBio Sequel	PacBio
Assembly method	Hifiasm	Falcon_UNZIP v. 0.7.0	Hifiasm v. AUGUST-2023	Mabs v. 2.24	FLYE v. 2.9.1	MaSuRCA v. 4.0.5; nextDenovo v. 2.5.0	Mabs v. 2.2	HiFiasm v. MAY-2022
Busco								
Single copy (%)	62.0	95.4	90.6	92.8	N/A	N/A	N/A	N/A
Duplicated (%)	35.4	3.9	7.5	5.5	N/A	N/A	N/A	N/A
Fragmented (%)	0.7	0.2	0.2	0.4	N/A	N/A	N/A	N/A
Missing (%)	1.9	0.5	1.7	1.3	N/A	N/A	N/A	N/A

To improve genome contiguity, we applied an intermediate scaffolding step using the reference genome of *A. sierrensis* (GenBank accession ID GCA_044231785.1) and subsequently performed manual curation of the scaffolded assembly using *Juicebox *[16]. This process included the introduction of 44 breaks across 40 contigs. Importantly, this manual curation step resulted in a final assembly constructed exclusively from *A. triseriatus* chromosomal interactions.

As in other mosquito species, the *A. triseriatus* genome comprises three pairs of chromosomes [40], with no differentiated sex chromosomes and sex-determination alleles linked to homomorphic autosomes [41]. Of the 2.46 Gb total assembly, 2.23 Gb were organized into the three chromosomes, while the remaining ~ 230 Mb could not be reliably positioned within the chromosomal assemblies and were retained as unplaced sequences. Based on the assembled genome, the estimated chromosome sizes of *A. triseriatus* were 920, 740, and 570 Mb for chromosomes 1, 2 and 3 respectively (Fig. 1).

The assembly achieves chromosome-sized scaffolds supported by Hi-C data and manual curation (N50 = 427 Mb, *n* = 3; Table 2). Signals consistent with putative centromeric regions are observable in the heatmap (green circles; Fig. 1B). In addition, 70% of the genome was repeat-masked, with approximately 30% of the masked content matching known repeat sequences and the remainder consisting of simple or currently unidentified repeats. BUSCO analysis indicated 97.4% complete (single copy: 62.0%; duplicated: 35.4%), demonstrating both the completeness of the assembly and redundancy in gene content.

We identified 862 genes in *A. triseriatus* that were duplicated relative to other species and located within 20 kb of one another, a distance chosen to match the mean long-read length in our dataset (Suppl. Table 2). After excluding genes present in more than two copies, we retained 444 genes corresponding to 222 duplicate pairs. Among these, 192 duplicate pairs (384 genes) were supported by at least one long-read spanning both gene copies, with a total of 1,242 PacBio reads providing evidence for these duplicate structures. Our PacBio data also revealed multiple instances of tandemly duplicated genes within single reads, as shown in a representative example (Suppl. Figure 3).

### Horizontal gene transfer (HGT) acquired genes

We investigated *A. triseriatus* genes potentially acquired through horizontal gene transfer (HGT) [37–39]. Among the predicted *A. triseriatus* genes, six are putatively derived from HGT and their products exhibit homology to proteins of diverse origins (Table 3): three viral, two fungal, and one of mixed viral/plant origin.

**Table 3:** Horizontal gene transfer identification in Ades triseriatus genome

Gene	Alien index	HGT index	Best outgroup hit	Taxa outgroup hits	Notes
AT12395	15.634512	76	RVW97177.1	Virus/Plant	Retrovirus-related Pol polyprotein from transposon RE1
AT18553	30.0525663	117	UYL94370.1	Virus	Polyprotein
AT10111	27.2828462	84	QHA33696.1	Virus	Gag-Pol polyprotein
AT12872	26.8601209	103	XP_067787577.1	Fungi	Reverse transcriptase
AT01941	10.49485	62	KAL2293652.1	Fungi	Hypothetical protein
AT05339	21.3251389	92	UYL94370.1	Virus	Polyprotein

### Mitogenome

**Fig. 2:**
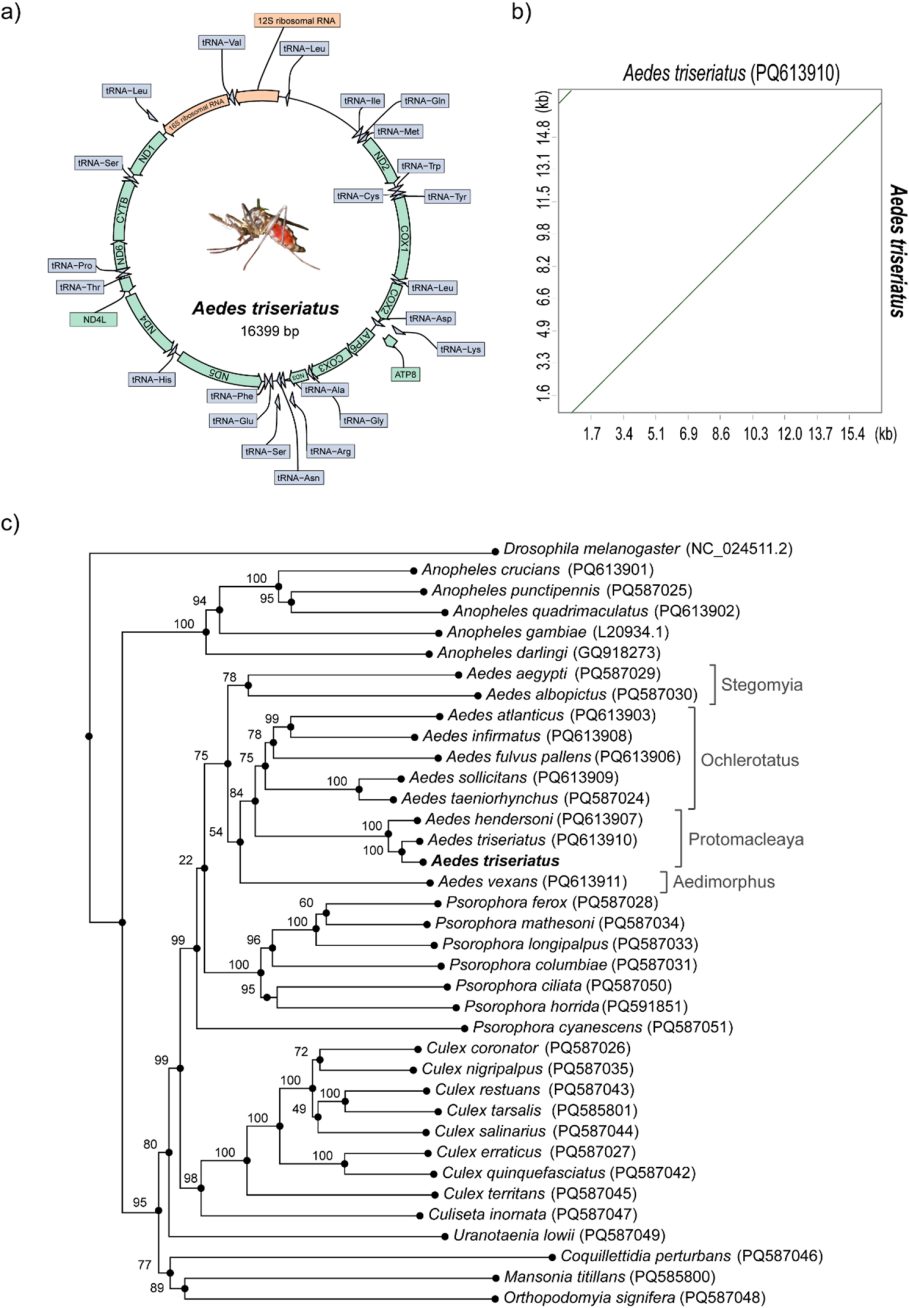
*Aedes triseriatus* mitogenome. **a** Structural representation of the circular mitogenome including length, orientation, and arrangement of the identified genes. **b** Mitogenome alignment between colony (bolded) and field-collected (PQ613910) strains. **c** Phylogeny mosquitoes including the *Aedes triseriatus* clade among other mosquito species

From our sequencing data, we assembled and annotated a complete circular *A. triseriatus* mitochondrial genome, representing the first report from a laboratory colony. The mitogenome contained a usual structure with 38 genes, including 13 protein-coding genes (PCGs), 22 tRNAs, and 2 rRNAs (Fig. 2a; Suppl. Table 4). Phylogenetic analysis confirmed that the sequence from our colony grouped with the recently published mitogenome from field-collected *A. triseriatus* (Fig. 2b and c), although the assembled length was slightly shorter (16,399 bp vs. 17,147 bp).

### Protein-coding genes annotation and expression values

Overall, we identified 21,240 unique genes, 43,739 transcripts, and 40,402 protein-coding sequences (CDS) (Suppl. Table 5). Functional gene annotations were assigned based on sequence similarity to three mosquito species (*A. aegypti*,* A. albopictus*, and *C. quinquefasciatus*). Homologous sequences from *Drosophila melanogaster* (FlyBase: https://flybase.org) and UniProt accession numbers (http://uniprot.org) were also considered to refine annotations and facilitate cross-species comparisons.

Among the predicted transcripts, 3,073 were not supported by detectable RNA reads. These may represent transcripts expressed at very low levels, in a few specialized cells, or corresponding to genes expressed during the non-covered embryonic developmental stage. Alternatively, they may correspond to annotation artifacts or nonfunctional loci such as pseudogenes.

**Fig. 3:**
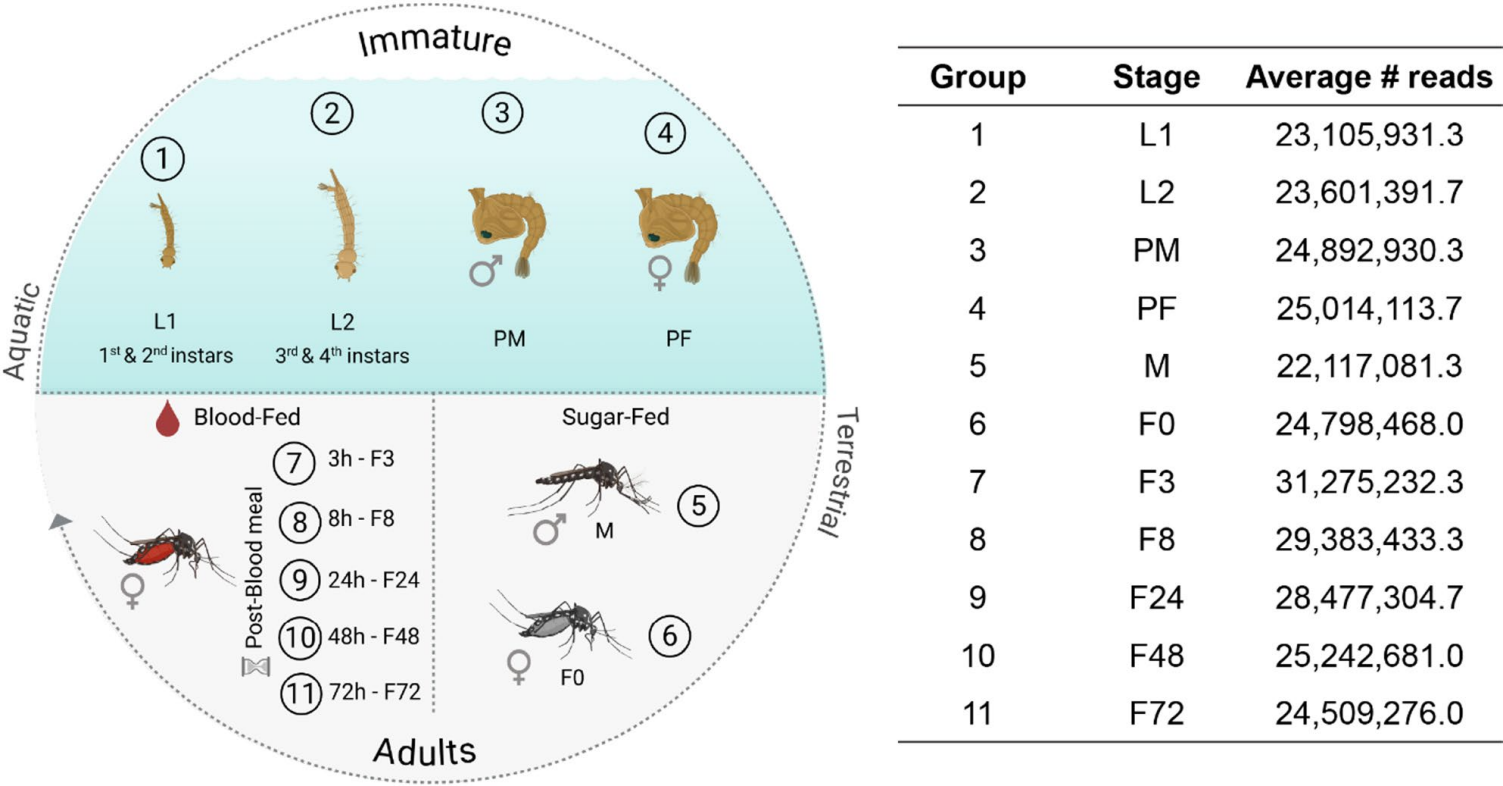
Schematic experimental design of the life stages and timepoints used in gene expression analysis of *A. triseriatus* and the average number of reads obtained in the biological triplicates of RNA-seq analysis

**Fig. 4:**
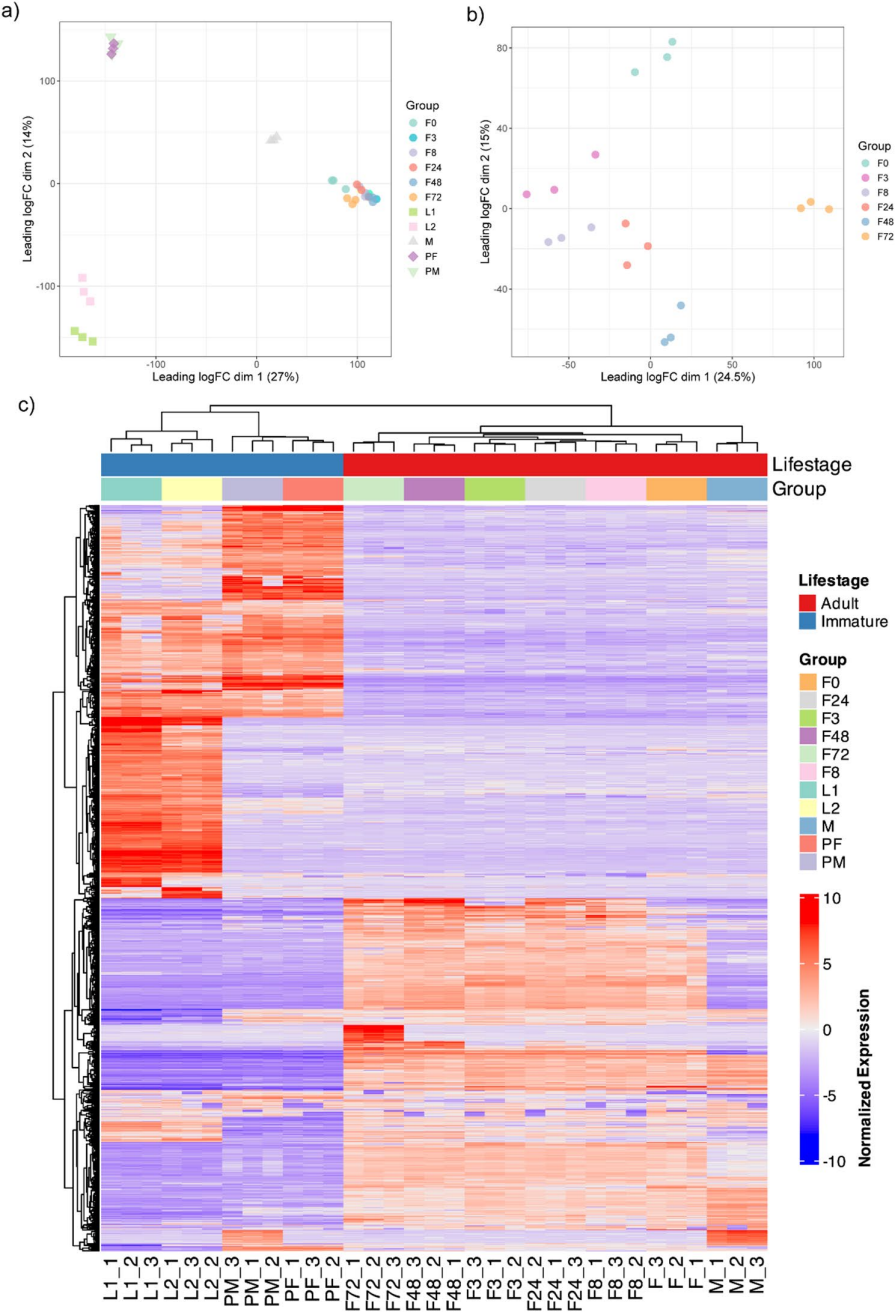
Multidimensional scaling (MDS) plot of *A. triseriatus* RNA-seq data: **a** all developmental stages, **b** Only blood-fed females, **c** Heatmap plot revealing differential gene expression across *Aedes triseriatus* lifecycle. Intensities are expressed as z-scores of the mean normalized TPM values (*n* = 3) of the transcripts identified across the mosquito life stages

To establish a reference dataset for future studies on *A. triseriatus* biology, we investigated gene expression across multiple developmental stages, including larvae, pupae, and adult males and females. Additionally, blood-fed females were evaluated at 3, 8, 24, 48, and 72 h post-feeding (Fig. 3). Our findings evidenced qualitative and quantitative transcriptional patterns (Fig. 3). The clear clustering of replicates by life stage and timepoint after the blood-meal in the MDS (Fig. 4a and b, respectively), and heatmap plot (Fig. 4c) demonstrates the reproducibility and consistency of gene expression profiles.

In total, 43,739 transcripts were identified, 14,567 of which are putative novel transcripts coding for proteins of unknown function (Table 4). Among all transcripts, 2,310 were identified as stage-specific markers, defined as transcripts with ≥ 95% of their expression restricted to a single developmental stage (Table 4). Of these, 1,060 lacked similarities to predicted proteins. About half of those are expressed in adult females (*n* = 604), suggesting potential roles in blood-feeding, reproduction and host seeking behavior. These findings provide a framework for understanding developmental regulation and identifying novel molecular targets for mosquito control.

**Table 4:** Transcript distribution across developmental stages of *Aedes triseriatus* mosquito.

Developmental Stage	Stage-specific transcripts (*n*)	Stage-specific Transcripts of unknown function [*n* (% total)]
L1	278	119 (42.8%)
L2	235	114 (48.5%)
PM	260	124 (47.7%)
PF	193	97 (50.3%)
M	192	102 (53.1%)
F0	147	62 (42.2%)
F3	158	77 (48.7%)
F8	134	68 (50.7%)
F24	147	82 (55.8%)
F48	230	114 (49.6%)
F72	336	201 (49.6%)
Overall	N/A	14,567 (-%)

### Constitutively highly expressed and stage-specific expressed genes

A subset of *A. triseriatus* genes is expressed both abundantly and constitutively (Suppl. Tables 5-7). Among these, many encoded proteins involved in protein synthesis and modification, including ribosomal proteins (AT02025, AT03203), translation initiation factors (AT03860), elongation factors (AT04872), and components of transcription machinery such as RNA editing (splicing) factors (AT14670). Cytoskeletal proteins, including actin (AT00255), myosin (AT02628), and α-tubulin (AT06297), are also highly expressed, consistent with their essential roles in cellular organization. Polyubiquitin (AT01834) is also expressed at high levels in these mosquitoes, encoding proteins that participate in diverse cellular processes such as chromatin structure modification, DNA repair, cell cycle regulation, protein degradation, and stress response. Mitochondrial proteins (AT06400, AT03989) are also among the most highly expressed genes.

Genes exhibiting stage-, sex-specific, or enhanced expression patterns demonstrate the dynamic regulation of gene expression throughout insect development. For example, those encoding Hexamerins (AT08292, AT09983, AT11848, AT18602, AT21804), are highly expressed during larval development and their encoded proteins serve as reservoirs for energy and amino acids during metamorphosis. Genes upregulated in females following a blood meal include those involved in egg production, such as yolk proteins (Vitellogenins: AT01412, AT01405; Vitellogenin receptor: AT03158), genes associated with yolk degradation (Vitellogenic carboxypeptidase: AT12514), and genes encoding eggshell components (Chorion peroxidase AT03072; Vitelline membrane protein: AT07224). Beta tubulin (AT14889), a marker of meiosis and sperm maturation, and the Sperm-Leucyl aminopeptidase (AT21185) are preferentially expressed in males.

Salivary secretions are essential for hematophagous mosquitoes, facilitating blood feeding by disrupting host defenses such as coagulation, platelet aggregation, vasoconstriction, and inflammation [42]. Earlier studies described the *A. triseriatus* sialome using Sanger sequencing and later Illumina-based approaches [43]. Here, we confirm and extend those findings using updated gene and transcript annotations (Suppl. Tables 5-7). Among the already known salivary proteins are D7s [44](encoded by AT21254, AT20525, AT08003, AT14851, AT14853, AT19582, AT14851, AT14852, AT19582), apyrases [45] (AT02738, AT16339, AT13390, AT02745), serpins [46] (AT16768, AT10161, AT10161, AT21168, AT06506, AT12583, AT10773, AT16682, AT11548), SGS [47] (AT14945, AT06123, AT06124, AT14944, AT18739), aegyptin [48] (AT14268, AT16245), among others.

Odorant-binding proteins (OBPs) are a highly abundant class of secreted proteins in the mosquito olfactory system, playing key roles in chemosensory pathways by binding, solubilizing, and transporting semiochemicals (odorants, pheromones, and sapid molecules) through the sensillum lymph to their receptors for signal transduction [49]. OBPs are primarily associated with mating, host-seeking, eggshell formation, and oviposition [50, 51].

In *Aedes triseriatus*, we identified 82 highly expressed odorant-binding protein (OBP) coding sequences (CDSs) (representing a relative low number of OBPs among major vector species when compared with *Anopheles gambiae* (69), *C. quinquefasciatus* (109), and *A. aegypti* (116) [52]. These findings align with a previous investigation of the *A. triseriatus* sialome [12], which reported 13 transcripts expressed at low levels.

In this study, six OBPs were detected exclusively in adult females, and were preferentially expressed 72 h after a blood meal (F72), suggesting potential role in oviposition or eggshell formation (AT07057, AT09978, AT11617, AT13784, AT15383, AT21454). Additionally, twelve OBPs showed preferential expression in larval stages (L1 and L2). Although their biological function remains unclear, we speculate that they may be involved in sensing predators or environmental toxins, which represent unfavorable conditions for development and could influence larval maturation.

Nevertheless, the presence of homologous genes across mosquito taxa does not necessarily imply conserved function, as exemplified by the D7 family, where orthologous proteins display divergent, species-specific activities [53]. Functional and biochemical assays therefore will be required to determine whether the newly identified *A. triseriatus* homolog proteins retain similar activities to those in their counterpart species. This warning also applies more broadly to all functional assignments in this study, as well as other works, that are based solely on sequence similarity.

A comprehensive description of the large number of genes regulated at each analyzed time point, along with the biochemical pathways or biological processes in which they participate, lies beyond the scope of this article. Instead, we highlighted only a few genes or groups of genes that show high levels of expression or major differences in transcript accumulation during the mosquito life cycle (Suppl. Table 7). These genes with well-established expression profiles in other mosquitoes support the soundness of our data [54, 55]. For more detailed exploration, a searchable database is available (Suppl. Tables 5 and 6). This resource allows investigators to perform targeted analyses of specific gene groups according to their functions, annotations, or expression patterns, tailored to their own research needs.

## Discussion

The projected large genome size confirmed by the cutting-edge technologies used in this study is supported by previous estimates of 1.5 and 2.1 Gb based on Feulgen cytophotometry and reassociation kinetics-based experiments, respectively [56, 57], which are considered less precise techniques when compared to contemporary sequencing approaches.

The initial contigs lacked the structure, organization, and orientation required to reconstruct chromosome-scale scaffolds. To improve contiguity, we applied an intermediate scaffolding step using the reference genome of *A. sierrensis*, a related species predominant along the U.S. West Coast and commonly known as Western tree hole mosquito [58]. Because the *A. sierrensis* genome itself was scaffolded against the *Aedes aegypti* reference genome, this approach could potentially introduce bias and propagate reference-associated errors. To mitigate such error propagation and to generate a chromosome-scale assembly supported by Hi-C that more accurately reflects the intrinsic chromosomal structure of *Aedes triseriatus*, we performed manual curation, rendering the final genome assembly effectively comparable to a *de novo* assembly rather than a reference-guided one.

Certain structural features, including definitive centromere placement and robust inter-arm interaction signals, could not be fully resolved. These limitations likely reflect the highly repetitive and complex nature of pericentromeric regions, which are inherently challenging to assemble and scaffold accurately, as reported in other mosquito genome studies [59]. Importantly, we did not observe abrupt interaction discontinuities, conflicting interaction blocks, or cross-chromosomal contact patterns that would indicate large-scale scaffold misjoins.

Despite repeat masking, the assembly retained a notably high rate of sequence duplication. Consistent with our observations, previous studies estimated that the genome of *A. triseriatus* contains a large proportion (85%) of repetitive DNA, most of it highly repetitive [56]. The complex Hi-C interaction pattern further supports the abundance of repetitive sequences and the high duplication rate. The clear primary diagonal indicates strong internal consistency and supports the biological validity of the detected intra-chromosomal interactions.

Although an elevated proportion of duplicated genes can sometimes reflect assembly artifacts, comparable duplication levels (32.7%) were reported in a recent independent assembly of the *A. triseriatus* genome from field-collected specimens [60]. Moreover, recent gene duplication events *in A. triseriatus* have been confirmed, including duplications of the Nix gene [41] and genes coding salivary Glycine-Rich Polypeptides [43], support the biological contribution of to the observed redundancy.

In summary, the genome organization and structural analysis of *A. triseriatus* reveal a complex architecture characterized by its large size and high level of sequence duplication. The integration of long-read sequencing and Hi-C scaffolding enabled the reconstruction of a highly contiguous chromosome-scale genome structure, with large scaffolds corresponding to the expected chromosome sizes, clear arm-level organization, and detectable signals consistent with putative centromeric regions. Although inter-arm interaction signals are less pronounced, likely reflecting the highly repetitive and heterochromatic nature of pericentromeric regions, the overall Hi-C patterns support the structural coherence of the assembly. While additional data or complementary approaches may further refine certain regions, this genome represents the most complete and contiguous resource currently available for *A. triseriatus*.

Our mitochondrial genome assembly grouped with previously reported *A. triseriatus* genome, with minor differences in total length likely reflecting natural genetic diversity and/or differences in assembly or annotation strategies affecting repetitive sequences [61]. These results provide a reference framework for evaluating intraspecific variation, lineage fixation, and potential divergence during laboratory colony maintenance.

The comprehensive annotation of protein-coding genes and transcriptome profiling across developmental stages reveal extensive transcriptional specialization throughout the life cycle of *A. triseriatus*. The identification of stage-specific markers, female-enriched transcripts following blood feeding, and lineage-specific gene duplications underscores the dynamic regulation underlying development, reproduction, and host interaction. Predicted transcripts without detectable coverage in the transcriptome may also reflect artifactual predictions arising from the annotation process or represent nonfunctional loci such as pseudogenes.

Although functional assignments are based primarily on sequence similarity and will require experimental validation, the integrated genomic and transcriptomic framework presented here provides a valuable foundation for investigating vector competence, physiological adaptation, and host-pathogen interactions in this medically important species. These resources will support future functional studies and enable comparative analyses across mosquito taxa.

## Conclusions

In this study, we present a chromosome-scale genome assembly and developmental transcriptome of the medically important mosquito *Aedes triseriatus*, generated using an integrated approach that combines PacBio, Hi-C, and Illumina technologies. The structural characterization of the *A. triseriatus* genome highlights its substantial size and remarkable level of duplication, underscoring the complexity of its genomic landscape.

We generated a chromosome-scale genomic resource that captures the major features of chromosomal organization, including arm-level structure and regions consistent with centromeric domains. Although some long-range interactions across chromosomal arms remain attenuated, overall interaction patterns indicate a coherent large-scale genomic structure. While further refinement may be achieved with new approaches, the present assembly constitutes the most comprehensive genomic reference available for *A. triseriatus* to date.

Together, these resources establish a robust genomic framework for advancing molecular studies of La Crosse virus (LACV) transmission and the broader biology of this vector and can be revisited as new technologies continue to improve structural resolution. Ultimately, these findings have the potential to inform innovative strategies for vector control and contribute to reducing the burden of mosquito-borne diseases.

## Supplementary Information

Supplementary Material 1. Supplementary Table 1: Species included in the *Aedes triseriatus* mitogenome and phylogenetic analyses.

Supplementary Material 2. Supplementary Table 2: Distribution of long-read sequencing lengths for the *Aedes triseriatus* genome, summarized in 1 kb bins.

Supplementary Material 3. Supplementary Table 3: Duplicated genes identified in the *Aedes triseriatus* genome.

Supplementary Material 4. Supplementary Table 4: Gene composition and features of the *Aedes triseriatus* mitochondrial genome.

Supplementary Material 5. Supplementary Table 5: Functional annotation of the *Aedes triseriatus* genome.

Supplementary Material 6. Supplementary Table 6: Pairwise comparisons of differentially expressed genes in *Aedes triseriatus*.

Supplementary Material 7. Supplementary Table 7: Selected genes used in the analysis of constitutive and stage-specific gene expression in *Aedes triseriatus*.

## Data Availability

All read data and final assemblies have been deposited to National Institute for Biotechnology Information (NCBI) under Bioproject PRJNA1321336. The raw sequencing dataset generated for this project including Illumina and Pacbio data were submitted to the NCBI Sequence Read Archive (https://identifiers.org/insdc.sra) under the following accession numbers: Pacbio Revio reads (SRX32493910) and HiC reads (SRX32493911) used to scaffold the assemblies; and Illumina short-reads for the developmental stage dataset: SRP618093. Finally, custom R scripts used in horizontal gene transfer analysis are available at: https://github.com/liliancaesarbio/alien_hgt_index.

## Abbreviations

*A. aegypti*: *Aedes aegypti*
*A. albopictus*: *Aedes albopictus*
*A. sierrensis*: *Aedes sierrensis*
*A. triseriatus*: *Aedes triseriatus*
Bp: Base pairs
*C. quinquefasciatus*: *Culex quinquefasciatus*
CDS: Coding sequences
Gb: Gigabases
gDNA: Total genomic DNA
HGT: Horizontal gene transfer
Hi-C: High-throughput Chromosome Conformation Capture
ID: Identification
Kb: Kilobases
LACV: La Crosse virus
Mb: Megabases
MDS: Multidimentional scaling
NR: Non-redundant
PCG: Protein-coding genes
rRNA: Ribosomal RNA
TPM: Transcripts per million
tRNA: Transfer RNA
VB: VectorBase database
