# Supplementary figures and images for: Chromosome-scale genome assembly and developmental transcriptome of Aedes triseriatus, vector of La Crosse virus
Source: BMC Genomics. 2026 Mar 25;27:433. doi: 10.1186/s12864-026-12779-8 (PMC13137584; doi:10.1186/s12864-026-12779-8)

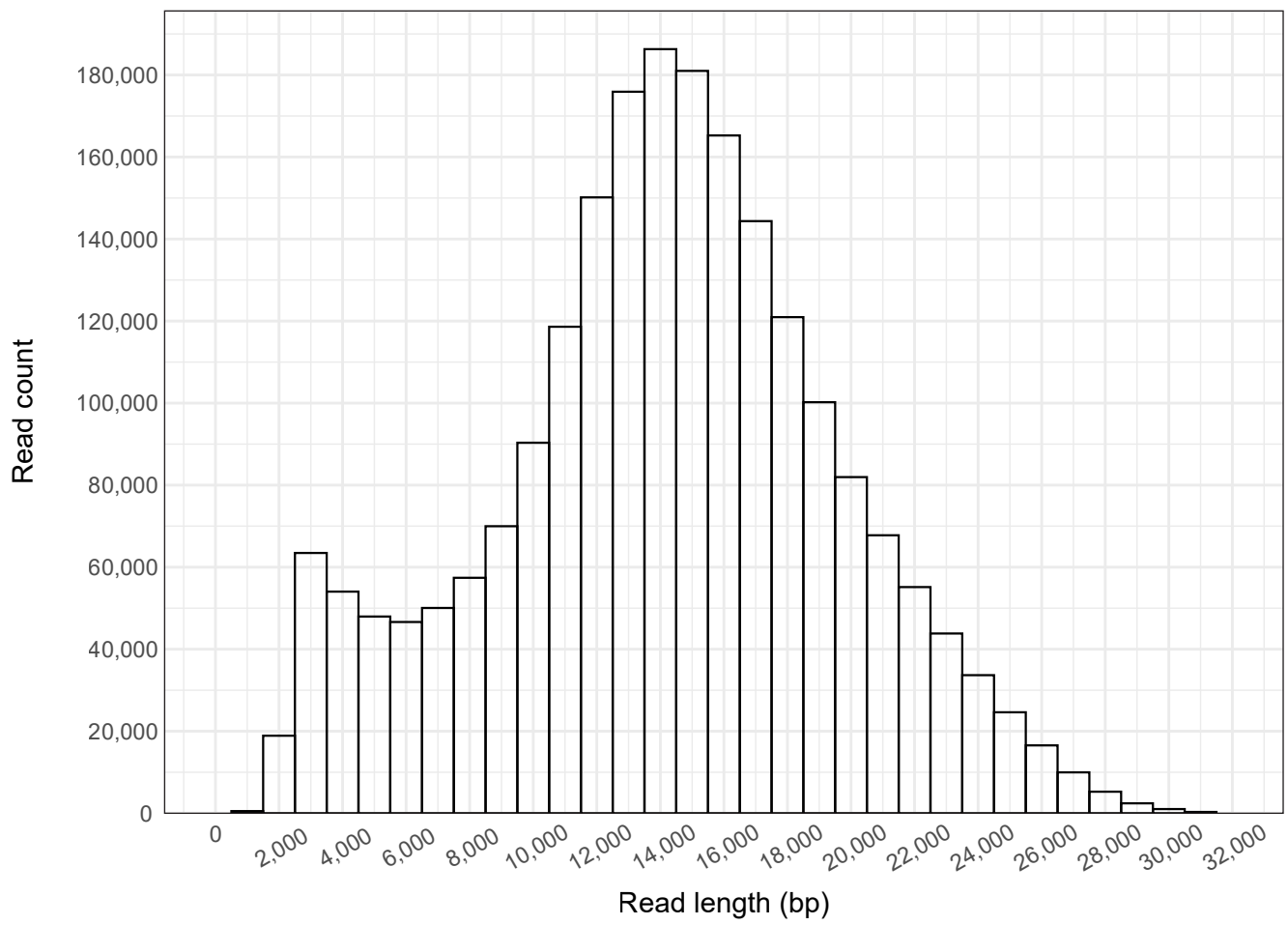

Supplement: Supplementary file 1 — Supplementary Material 1. Supplementary Table 1: Species included in the Aedes triseriatus mitogenome and phylogenetic analyses. [file 12864_2026_12779_MOESM1_ESM.pdf]

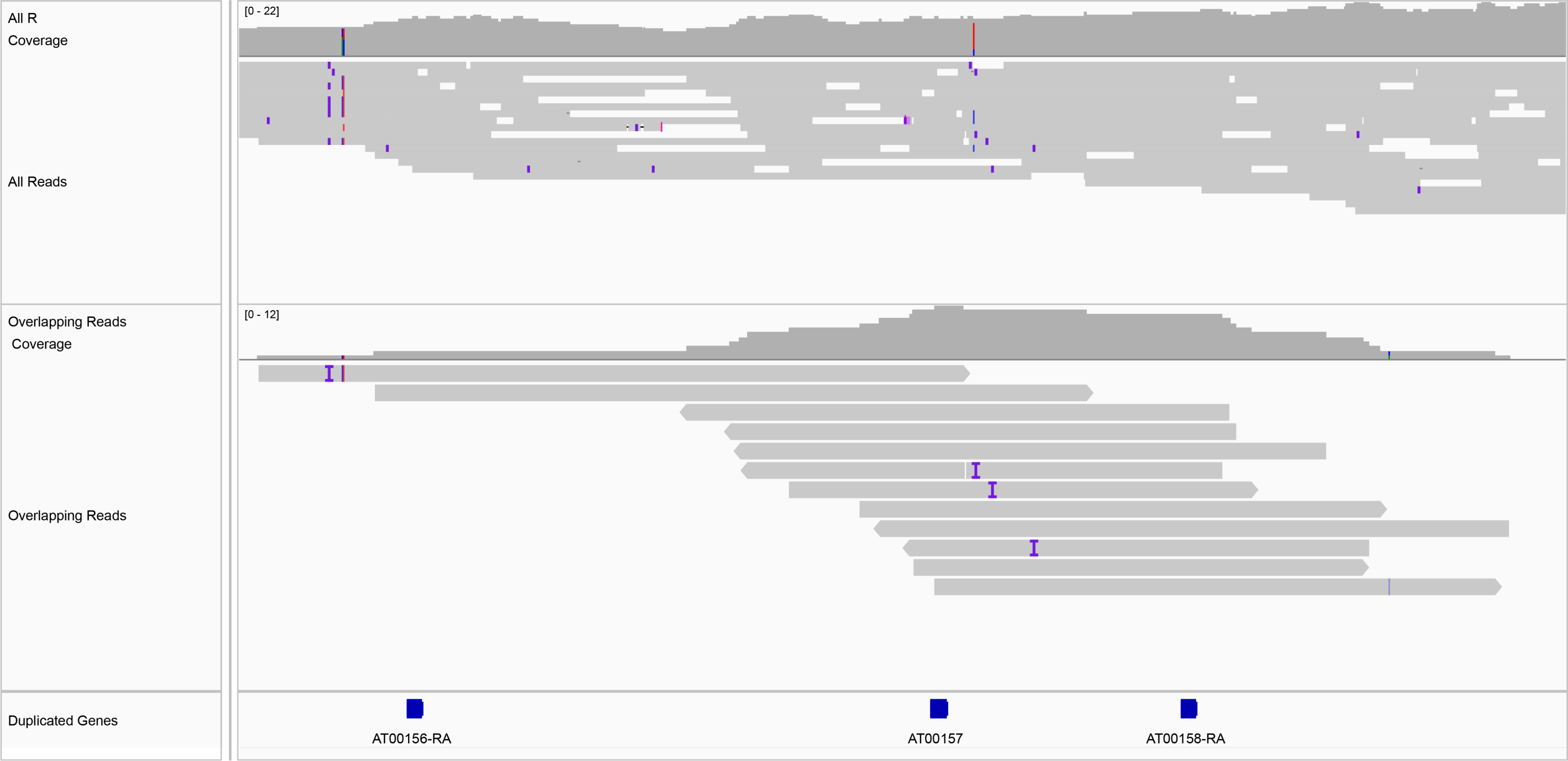

Supplement: Supplementary file 2 — Supplementary Material 2. Supplementary Table 2: Distribution of long-read sequencing lengths for the Aedes triseriatus genome, summarized in 1 kb bins. [file 12864_2026_12779_MOESM2_ESM.pdf]
