# Supplementary material for: Chromosome-scale genome assembly and developmental transcriptome of Aedes triseriatus, vector of La Crosse virus
Source: BMC Genomics. 2026 Mar 25;27:433. doi: 10.1186/s12864-026-12779-8 (PMC13137584; doi:10.1186/s12864-026-12779-8)
Supplement: Supplementary file 3 — Supplementary Material 3. Supplementary Table 3: Duplicated genes identified in the Aedes triseriatus genome. [file 12864_2026_12779_MOESM3_ESM.docx]

# **Supplementary** **Data**

Suppl.Table 1: Distribution of long-read sequencing lengths for the Aedes triseriatus genome, summarized in 1 kb bins.

| Read length interval (bp) | Read count | Percentage of reads (%) |
| --- | --- | --- |
| 0-1,000 | 105 | 0.0 |
| 1,000-2,000 | 3671 | 0.2 |
| 2,000-3,000 | 46758 | 2.1 |
| 3,000-4,000 | 60731 | 2.8 |
| 4,000-5,000 | 49938 | 2.3 |
| 5,000-6,000 | 46808 | 2.1 |
| 6,000-7,000 | 47873 | 2.2 |
| 7,000-8,000 | 53218 | 2.4 |
| 8,000-9,000 | 62875 | 2.9 |
| 9,000-10,000 | 79091 | 3.6 |
| 10,000-11,000 | 103459 | 4.7 |
| 11,000-12,000 | 134609 | 6.2 |
| 12,000-13,000 | 164835 | 7.5 |
| 13,000-14,000 | 183125 | 8.4 |
| 14,000-15,000 | 184966 | 8.5 |
| 15,000-16,000 | 174102 | 8.0 |
| 16,000-17,000 | 155083 | 7.1 |
| 17,000-18,000 | 133011 | 6.1 |
| 18,000-19,000 | 109973 | 5.0 |
| 19,000-20,000 | 90832 | 4.2 |
| 20,000-21,000 | 74576 | 3.4 |
| 21,000-22,000 | 61221 | 2.8 |
| 22,000-23,000 | 49246 | 2.3 |
| 23,000-24,000 | 38843 | 1.8 |
| 24,000-25,000 | 28835 | 1.3 |
| 25,000-26,000 | 20479 | 0.9 |
| 26,000-27,000 | 13107 | 0.6 |
| 27,000-28,000 | 7345 | 0.3 |
| 28,000-29,000 | 3599 | 0.2 |
| 29,000-30,000 | 1584 | 0.1 |
| 30,000-31,000 | 600 | 0.0 |
| 31,000-32,000 | 206 | 0.0 |

Suppl.Table 3: Species used in the mitogenome and phylogenetic tree analysis.

| # | Species | GenBank Reference Sequence |
| --- | --- | --- |
| 1 | *Aedes aegypti* | PQ587029 |
| 2 | *Aedes albopictus* | PQ587030 |
| 3 | *Aedes atlanticus* | PQ613903 |
| 4 | *Aedes fulvus pallens* | PQ613906 |
| 5 | *Aedes hendersoni* | PQ613907 |
| 6 | *Aedes Infirmatus* | PQ613908 |
| 7 | *Aedes sollicitans* | PQ613909 |
| 8 | *Aedes taeniorhynchus* | PQ587024 |
| 9 | *Aedes triseriatus* | PQ613910 |
| 10 | *Aedes Vexans* | PQ613911 |
| 11 | *Anopheles crucians* | PQ613901 |
| 12 | *Anopheles darlingi* | GQ918273 |
| 13 | *Anopheles gambiae* | L20934.1 |
| 14 | *Anopheles punctipennis* | PQ587025 |
| 15 | *Anopheles quadrimaculatus* | PQ613902 |
| 16 | *Coquillettidia perturbans* | PQ587046 |
| 17 | *Culex coronator* | PQ587026 |
| 18 | *Culex erraticus* | PQ587027 |
| 19 | *Culex nigripalpus* | PQ587035 |
| 20 | *Culex quinquefasciatus* | PQ587042 |
| 21 | *Culex restuans* | PQ587043 |
| 22 | *Culex salinarius* | PQ587044 |
| 23 | *Culex tarsalis* | PQ585801 |
| 24 | *Culex territans* | PQ587045 |
| 25 | *Culiseta inornata* | PQ587047 |
| 26 | *Drosophila melanogaster* | Flybase release 6.54 |
| 27 | *Mansonia Titillans* | PQ585800 |
| 28 | *Orthopodomyia signifera* | PQ587048 |
| 29 | *Psorophora ciliata* | PQ587050 |
| 30 | *Psorophora columbiae* | PQ587031 |
| 31 | *Psorophora cyanescens* | PQ587051 |
| 32 | *Psorophora discolor* | PQ587032 |
| 33 | *Psorophora ferox* | PQ587028 |
| 34 | *Psorophora horrida* | PQ591851 |
| 35 | *Psorophora longipalpus* | PQ587033 |
| 36 | *Psorophora mathesoni* | PQ587034 |

Suppl. Table 4: Gene Composition and Features of the Aedes triseriatus Mitochondrial Genome.

| Molecule | Gene | Start | End | Strand | Orientation |
| --- | --- | --- | --- | --- | --- |
| h1tg002340l | trnM(cat) | 1 | 70 | forward | 0 |
| h1tg002340l | nad2 | 97 | 1009 | forward | 0 |
| h1tg002340l | trnW(tca) | 1097 | 1166 | forward | 0 |
| h1tg002340l | trnC(gca) | 1165 | 1232 | reverse | 1 |
| h1tg002340l | trnY(gta) | 1232 | 1298 | reverse | 1 |
| h1tg002340l | cox1 | 1302 | 2811 | forward | 0 |
| h1tg002340l | trnL2(taa) | 2833 | 2900 | forward | 0 |
| h1tg002340l | cox2 | 2901 | 3589 | forward | 0 |
| h1tg002340l | trnK(ctt) | 3586 | 3657 | forward | 0 |
| h1tg002340l | trnD(gtc) | 3673 | 3742 | forward | 0 |
| h1tg002340l | atp8 | 3742 | 3901 | forward | 0 |
| h1tg002340l | atp6_0 | 3897 | 4257 | forward | 0 |
| h1tg002340l | atp6_1 | 4264 | 4426 | forward | 0 |
| h1tg002340l | cox3 | 4438 | 5218 | forward | 0 |
| h1tg002340l | trnG(tcc) | 5220 | 5287 | forward | 0 |
| h1tg002340l | nad3 | 5314 | 5641 | forward | 0 |
| h1tg002340l | trnR(tcg) | 5639 | 5703 | forward | 0 |
| h1tg002340l | trnA(tgc) | 5738 | 5805 | forward | 0 |
| h1tg002340l | trnN(gtt) | 5806 | 5875 | forward | 0 |
| h1tg002340l | trnS1(gct) | 5873 | 5940 | reverse | 1 |
| h1tg002340l | trnE(ttc) | 5956 | 6022 | forward | 0 |
| h1tg002340l | trnF(gaa) | 6032 | 6099 | reverse | 1 |
| h1tg002340l | nad5 | 6154 | 7732 | reverse | 1 |
| h1tg002340l | trnH(gtg) | 7846 | 7912 | reverse | 1 |
| h1tg002340l | nad4 | 7940 | 9185 | reverse | 1 |
| h1tg002340l | nad4l | 9259 | 9517 | reverse | 1 |
| h1tg002340l | trnT(tgt) | 9555 | 9621 | forward | 0 |
| h1tg002340l | trnP(tgg) | 9621 | 9688 | reverse | 1 |
| h1tg002340l | nad6 | 9693 | 10209 | forward | 0 |
| h1tg002340l | cob | 10214 | 11333 | forward | 0 |
| h1tg002340l | trnS2(tga) | 11343 | 11409 | forward | 0 |
| h1tg002340l | nad1 | 11457 | 12357 | reverse | 1 |
| h1tg002340l | trnL1(tag) | 12384 | 12452 | reverse | 1 |
| h1tg002340l | rrnL | 12431 | 13729 | reverse | 1 |
| h1tg002340l | trnV(tac) | 13787 | 13859 | reverse | 1 |
| h1tg002340l | rrnS | 13858 | 14649 | reverse | 1 |
| h1tg002340l | trnI(gat) | 16259 | 16328 | forward | 0 |
| h1tg002340l | trnQ(ttg) | 16332 | 16399 | reverse | 1 |
